# Current indications for the intrathoracic transposition of the omentum

**DOI:** 10.1186/s13019-019-0924-9

**Published:** 2019-06-10

**Authors:** Petre V. H. Botianu

**Affiliations:** Surgery IV Discipline, M5 Department, University of Medicine and Pharmacy from Tirgu-Mures, 540091 Bujorului 2A, Tirgu-Mures, Romania

**Keywords:** Omentoplasty, Omentum, Mediastinitis, Median sternotomy, Eso-gastric anastomosis, Esophagectomy, Bronchial fistula, Space-filling procedures, Empyema, Mediastinal tracheostomy, Infected vascular grafts

## Abstract

**Background:**

The intrathoracic transposition of the omentum (ITO) has been reported with more or less good results in various clinical circumstances but with no clear guidelines or indications.

**Methodology and review:**

This article reviews the main clinical situations in which omento-plasty (OP) may be taken into consideration by the thoracic surgeons: mediastinitis and deep sternal infections after median sternotomy, reinforcement of the eso-gastric anastomosis after esophagectomy, prevention and treatment of the bronchial fistula after pulmonary resection, space-filling procedures for empyema, mediastinal tracheostomy, management of the infected intrathoracic vascular grafts / ventricular assist devices and heart OP. For each clinical situation we have performed a literature review with analysis of the most relevant published papers searching for an evidence-based approach for the use of the ITO/OP in thoracic surgery.

**Conclusions:**

OP may be an elegant solution for a wide range of problems in thoracic surgery. In the published literature, there are mainly case-reports and relatively small series published resulting in a low level of evidence for both ITO as a surgical technique by itself, as well as for the use of OP in various clinical situations involving the chest structures. The indications for its use in thoracic surgery are based more on common sense and the lack of other solutions.

## Background

The omentum is well known as having an excellent plasticity and blood supply and its use has been reported in a great variety of defects with good results [1, 2]. Although not very frequently used in thoracic surgery, omentoplasty (OP) may be an elegant solution for a wide range of problems, including the prevention and treatment of life-endangering complications [3, 4]. However, most of the published studies include relatively small series and for most of the clinical situations there are neither guidelines, nor prospective randomised studies to allow clear indications for its use in thoracic surgery. Since in most cases the available literature does not allow an evidenced-based approach due to the small number and the great heterogenicity of the patients, our aim was to provide an overview of the use of this specific surgical technique (OP) in the nowadays thoracic surgery practice. This paper presents and discusses the current indications for the intrathoracic transposition of the omentum (ITO), with specific considerations on the arguments and analysis of the available evidence for OP in certain clinical situations.

## Methodology

We have performed a literature review concerning the use of ITO / OP in thoracic surgery using the PubMed and Medline databases up to may 2018 using the following combinations of MeSH terms: [Omentum, Omentoplasty, Epiploon] AND [Mediastinitis / Deep Sternal Infections / Median Sternotomy], [Esophagectomy / Eso-Gastric Anastomosis], [Bronchial Closure / Bronchial Fistula / Pulmonary Resection / Lobectomy / Pneumonectomy], [Empyema], [Mediastinal Tracheostomy], [Infected Vascular Graft], [Ventricular Assist Device / VAD], [Heart].

## Review

We present a review concerning the indications and the main arguments for and against the use of the omentum flap (OF) in the following clinical situations: mediastinitis and deep sternal infections after median sternotomy, reinforcement of the eso-gastric anastomosis after esophagectomy, prevention and treatment of the bronchial fistula after pulmonary resection, space-filling procedures for empyema, mediastinal tracheostomy, management of the infected intrathoracic vascular grafts / ventricular assist devices and heart OP. An emphasys was put on the level of evidence for using OP in each of the aforementioned clinical situations.

**Mediastinitis and deep sternal infections after median sternotomy** are an excellent indication for OP, which may be used alone or together with other flaps (usually pectoralis), with or without skin grafts, in order to achieve complete and definitive wound healing [5–8]. In a meta-analysis of 16 observational studies including 1046 patients treated in units using both OF and muscle flaps for the treatment of mediastinitis / deep sternal infections, van Wingerdeen et al. (2011) found that there is no evidence to prove the superiority of reconstruction with muscle flaps versus a laparotomy-harvested OF, while the use of the omentum may be associated with a lower mortality and fewer complications [9]. The OF can be easily mobilized using a small upper midline laparotomy and brought to fill the anterior mediastinum after debridement and/or the use of negative pressure wound therapy. The possibility to mobilize the OF using a laparoscopic approach makes this flap even more attractive by avoiding the morbidity associated with laparotomy [10]. A specific but rare complication of OP for mediastinitis and deep sternal wound infections is the possibility to develop a retrosternal herniation of the abdominal viscera through the diaphragmatic defect; reoperation and treatment of this particular hernia is possible without compromising the OF [11].

**Fig. 1:**
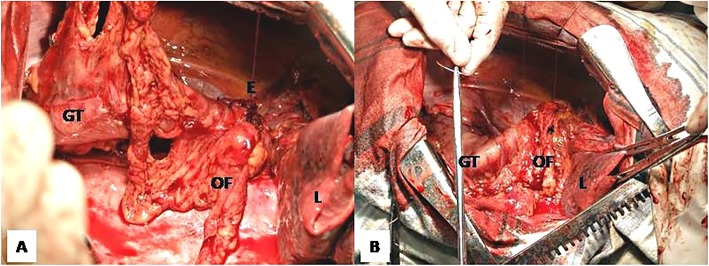
Intrathoracic eso-gastric anastomosis reinforced by omental wrapping. Note that the OF is mobilized together with the greater curvature gastric tube (**a**) and then wrapped around the eso-gastric anastomosis (**b**) which will be completely covered by the OF. (GT – gastric tube, E – esophagus, L – lung)

**Reinforcement of the eso-gastric anastomosis after esophagectomy** is a relatively new but increasingly popular indication for the ITO. This technique involves the mobilization of a relatively small OF based on the gastro-epiploic arcade and the epiploic vessels together with the greater curvature of the stomach (Fig. 1 a), which is then used to wrap the anastomosis between the gastric tube and the upper esophageal stump (Fig. 1b). It may be used to reinforce both intrathoracic [12] and cervical [13] eso-gastric anastomosis. There are several published studies showing that this technique is associated with a significant reduction of the incidence - 2.9% vs 10.5%, POR = 0.28, 95% CI = 0.17 to 0.47 and *P* < 0.0001 in the meta-analysis published by Wiggins in 2015, as well as the severity of the anastomotic fistulas [14], although it does not seem to reduce the overall mortality after esophagectomy [14–16]. Since this particular OF can be mobilized very easy and quick together with the gastric conduit without any additional morbidity, we may conclude that its use should be taken into consideration as a routine measure to reduce the morbidity after esophagectomy.

Quite interesting, we found no studies concerning the use of the OF to reinforce anastomoses after other types of esophageal reconstruction.

### Prevention and treatment of the bronchial fistula after pulmonary resection

**Fig. 2:**
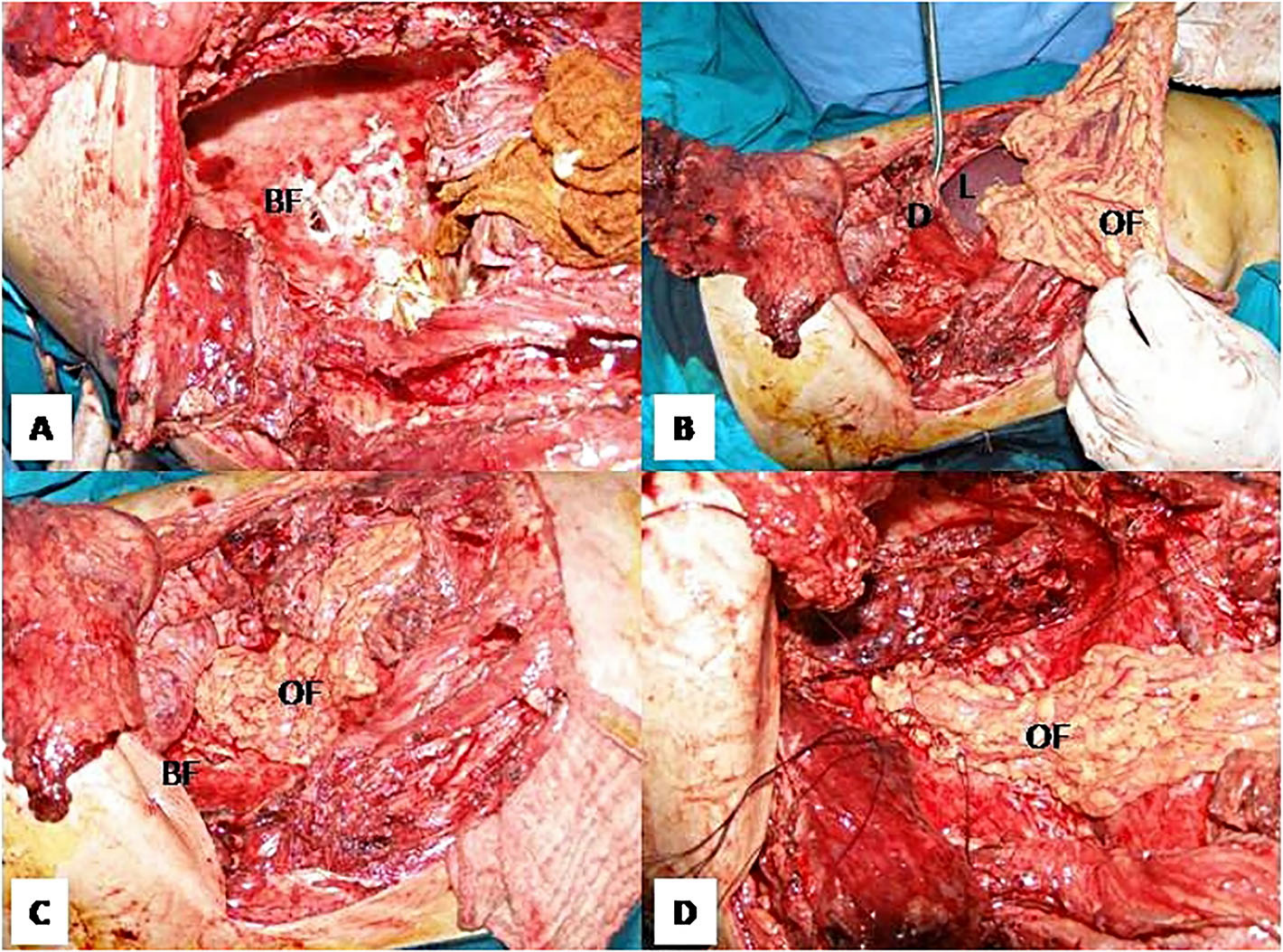
Closure of a large bronchial fistula after right pneumonectomy during a space-filling procedure (**a**). The OF was mobilized through a phrenotomy (**b**) and fxed with separate stitches over the bronchial fistula (**c**, **d**) without performing a supplementary laparotomy (BF – bronchial fistula, D – diaphragm, L - liver)

Reinforcement of the bronchial stump after lung resection is usually indicated in patients considered at high-risk for developing a bronchial fistula [17, 18] but the OF is rarely used in this situation since there are other more simple solutions not requiring an additional access to the abdominal cavity [19–21]. However, when a bronchial fistula develops after a pulmonary resection the use of an OF should be strongly taken into consideration if other less invasive solutions are not possible or have failed – usually after right pneumonectomy. The OF with its excellent vascularisation and plasticity allows a very easy and safe closure of the bronchial opening, even in the presence of active infection and fibrotic tissues (Fig. 2); there are several published papers with good results reported in small series of patients [22, 23] but we found no study comparing OP with other bronchial fistula closure techniques. Large airway defects not amenable to direct closure may be solved by the use of bioprosthetic materials covered by well-vascularised flaps – including the OF [24].

One of the classic main disadvantages of ITO/OP for both bronchial stump reinforcement and bronchial fistula closure is the need to perform a supplementary laparotomy, which requires to reposition the patient on the operating table, adds a significant increase of the operating time and is associated with a supplementary specific morbidity. D’Andrilli and colleagues recommend the transdiaphragmatic mobilization of the OF through the usual thoracotomy. Since closure of a bronchial fistula does not require a large volume flap, only a limited mobilization is usually required, thus involving a minimal dissection which may be performed safe and easy through a limited phrenotomy in the absence of peritoneal adhesions [25].

**Space-filling procedures for empyema** may benefit from the use of an OF, although its use in this condition involves a supplementary abdominal septic risk while most of the cases can be solved by thoracomyoplasty using neighbourhood muscle flaps [21, 26–29]. According to Le Fourn (1994) the presence of a bronchial fistula which may be closed using an omental patch, previous radiotherapy and a large posterolateral thoracotomy with sectioning of the latissimus dorsi are arguments to preferr OP [30]. Since the OF does not have a very large volume, it is necessary in most cases to add the use of other muscle flaps and/or a thoracoplasty in order to achieve a complete obliteration of the infected space [31, 32]. To note that the OF is frequently used for both the closure of the bronchial fistula and filling of the empyema cavity, alone or together with other muscle flaps while many of the studies dedicated to the space-filling procedures for empyema include cases solved by OP without clearly explaining the reason why the omentum was preferred over other flaps [33–37]. Intraparenchymatous infected cavities are nowadays extremely rare indications for space-filling procedures [38] while the omentum is difficult to mobilize inside a pulmonary cavity; in the available literature we found only case-reports with free OF transferred using microsurgery techniques for solving intrapulmonary infectious lesions [39].

### Mediastinal tracheostomy

During this rarely performed procedure – usually indicated for advanced neck cancers, OP has been reported to prevent vascular erosion with fatal bleeding. Most series include a small number of patients with no comparison between OP versus pectoralis muscle flap [40, 41].

### Management of the infected intrathoracic vascular grafts

This situation is known as a very difficult one with a poor outcome due to the association of sepsis and bleeding in patients with significant comorbidities. There is no standardized treatment with most cases requiring a multidisciplinary approach which includes more pharmacological, endovascular and open surgical options [42].

The OF is used for both wrapping the graft and filling the dead/infected mediastinal spaces, combined with other anti-infectious strategies. In selected cases, the in situ preservaton of the infected graft is possible by a combination of systemic and local antiinfectious therapy, local debridement and wrapping with well-vascularised flaps – including OP [43]. In the available literature we found only case-reports and small series, the largest single-center study dedicated precisely to this subject being the one published by Shah et al. in 2013 who reported only one death in a series of 11 patients with infected aortic grafts treated by OP [44]. In a multi-center retrospective study on 58 patients with infected intrathoracic vascular prosthesis, Oda et al. (2015) found that the use of muscle or OF to wrap the infected grafts was associated with a reduction of the mortality but no comparative study OP versus muscle flaps was performed [45].

A particular situation is the development of an aorto-esophageal fistula, which has a very high immediate fatal outcome potential while surgical repair is extremely challenging due to the complexity of the lesions and the presence of infection. The most succesfull strategy seems to be the one proposed recently by Nakamura and colleagues (2017) consisting in emergency graft replacement of the ruptured/perforated aorta and esophagectomy with mediastinal debridement to achieve local control of sepsis, as well as OP in order to protect the vascular graft which is implanted in a septic area [46]. Endovascular repair may allow an immediate temporary controle of the bleeding, which is usefull in the patients with haemorrhagic shock while an esophageal reconstruction in a later stage is possible if there are no other complications and the biological status of the patient allows another major procedure [46–48].

Prophylactic omental wrapping after the excision of infectious thoracic aorta aneurysms has been reported with good results in small series of patients [49–51]. In a series of 22 patients with infectious thoracic aortic aneurysms treated by excision and in situ grafting, Yamashiro et al. report better results in the patients with omental wrapping of the graft: with versus without omental wrapping operative mortality of 12.5 versus 50.0%, (*p* = 0.06) while the 5-year event-free survival rates were 84.6 versus 33.3% (*p* = 0.025). However, these results should be interpretated with caution due to the small number of the patients involved [50].

**Management of the infected ventricular assist devices** used for end-stage heart diseases has been recently reported to benefit from OP in small series. The OF is used to wrap the pump and connection tubes, as well as to fill the infected mediastinum and/or the spaces surrounding the ventricular assist devices [52, 53]. Considering the increasing use of the ventricular assist devices in the treatment of end-stage heart diseases this may be an important indication for the use of the OF in the near future.

**Heart omentopexy/OP** in order to induce myocardial neovascularisation is an old idea. It was used as a surgical solution for ischemic heart disease before the development of modern cardiac surgery [54] but it was abandoned due to both poor results and the development of more efficient methods for treating coronary atherosclerosis [55]. Some recent experimental studies showed promising results by using hybrid strategies involving heart omentopexy/OP to enhance the myocardial revascularisation induced by various cell therapies [56, 57] but no translation to human clinical practice has been performed yet.

## Conclusions

ITO/OP may be a solution for a wide range of problems in modern thoracic surgery. However, for most of the clinical situations analysed there is a low level of evidence to support its use and the indication to perform ITO/OP is based mainly on common sense and the lack of other alternatives, as well as a combination of easy mobilization and reduced associated morbidity. The possibility to perform the mobilization of the OF using a transdiaphragmatic [25] or laparoscopic [10, 58] approach may increase the indications for its use in the future. Thoracic surgeons should be familiar with the anatomy and the techniques of OF mobilization and ITO.

## Data Availability

Data sharing not applicable to this article as no datasets were generated or analysed during the current study.

## Abbreviations

ITO: intrathoracic transposition of the omentum
OF: omentum flap
OP: omentoplasty
